# Attenuated bidirectional short-term synaptic plasticity in the dentate gyrus of Schnurri-2 knockout mice, a model of schizophrenia

**DOI:** 10.1186/s13041-018-0400-9

**Published:** 2018-10-01

**Authors:** Katsunori Kobayashi, Tsuyoshi Takagi, Shunsuke Ishii, Hidenori Suzuki, Tsuyoshi Miyakawa

**Affiliations:** 1 Department of Pharmacology, Graduate School of Medicine, Nippon Medical School, 1-1-5 Sendagi, Bunkyo-ku, Tokyo, 113-8602 Japan; 2 Institute for Developmental Research, Aichi Human Service Center, Kasugai, Japan; 3 RIKEN Tsukuba Institute, Tsukuba, Japan; 4 Division of Systems Medical Science, Institute for Comprehensive Medical Science, Fujita Health University, Toyoake, Japan

**Keywords:** Short-term plasticity, Perforant path, Dentate gyrus, Schizophrenia

## Abstract

The dentate gyrus of the hippocampus has been implicated in the pathophysiological basis of neuropsychiatric disorders including schizophrenia. We have identified several mouse models of neuropsychiatric disorders with robust molecular and functional defects in the dentate gyrus. Among them, mice lacking Schnurri-2 (Shn2 or HIVEP2) have been proposed as a model of schizophrenia and intellectual disability. Shn2 knockout mice exhibit behavioral abnormalities resembling symptoms of schizophrenia and HIVEP2-related intellectual disability as well as marked functional alterations in the soma and output synapse of the dentate granule cells (GCs). Although robust abnormalities were also observed in the dendritic spine morphology in the GCs, their functional correlates remain unknown. In the present study, we performed electrophysiological analyses of synaptic transmission at the medial perforant path (MPP) input onto the GCs in Shn2 knockout mice. While the basal synaptic efficacy was preserved, short-term synaptic depression induced by paired-pulse or low-frequency stimulation was reduced in the mutant mice. High-frequency tetanic stimulation induced lasting synaptic potentiation in both wild-type and mutant mice. However, the decaying synaptic potentiation shortly after the tetanic stimulation was significantly reduced in the mutant mice. These results indicate that the Shn2 deficiency attenuates bidirectional short-term synaptic plasticity at the MPP-GC synapse, thereby rendering the synapse more static. Our finding further supports a possible role of the dentate gyrus dysfunction in pathophysiology of schizophrenia and may also provide important information in interpreting morphology changes of the brain synapses in neuropsychiatric disorders.

**Electronic supplementary material:**

The online version of this article (10.1186/s13041-018-0400-9) contains supplementary material, which is available to authorized users.

## Main text

The hippocampal dentate gyrus has been implicated in the pathophysiology of neuropsychiatric disorders including schizophrenia [1, 2]. In several mouse models of neuropsychiatric disorders, we have demonstrated shared molecular and functional defects in the dentate granule cells (GCs), characterized as the immature-like phenotype [3–6]. Among them, mice lacking Schnurri-2 (Shn2; also called major histocompatibility complex [MHC]-binding protein 2 [MBP-2], or human immunodeficiency virus type I enhancer binding protein 2 [HIVEP2]) have been proposed as a model for schizophrenia and HIVEP2-related intellectual disability with good face and construct validity [4, 7, 8]. Shn2 knockout mice exhibit behavioral abnormalities resembling symptoms of these disorders, mild brain inflammation, and transcriptome/proteome changes similar to those in the brain of schizophrenia [4]. At cellular levels, Shn2 knockout mice show marked changes in somatic firing properties of the GCs and greatly reduced activity-dependent facilitation at their output synapses [4]. We have recently examined subcellular-scale structures of the GCs of Shn2 knockout mice and revealed immature filopodia-like dendritic spines characterized by increased spine length and decreased neck diameter [7], suggesting possible functional changes in the excitatory synaptic input. Given potential importance of spine pathology in neuropsychiatric disorders [9], it would be important to examine functional correlates of altered spine morphology in these mice. Therefore, in the present study, we performed electrophysiological analyses of synaptic transmission at the medial perforant path (MPP) input onto the GCs in Shn2 knockout mice.

**Fig. 1:**
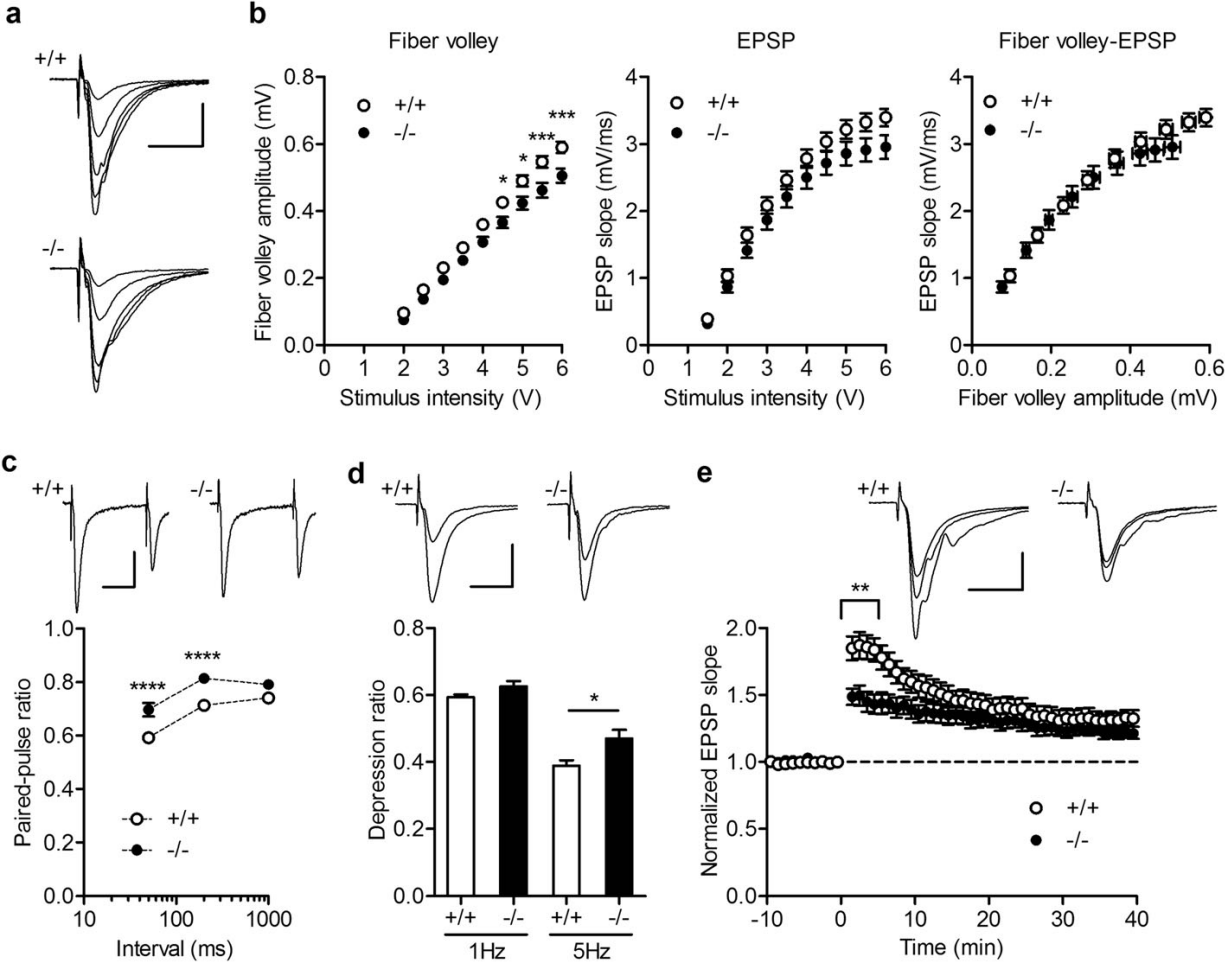
Impaired activity-dependent short-term synaptic plasticity in dentate granule cells of Shn2 knockout mice. **a** Medical perforant path (MPP) synaptic potentials evoked at the stimulus intensities of 1.5, 2, 3, 4 and 5 V in wild-type (+/+) and Shn2 knockout (−/−) mice. Scale bar: 10 ms, 1 mV. **b** Dependence of fiber volley amplitude (left) and EPSP slope (center) on stimulus intensity, and the relationship between fiber volley and EPSP (right). The mutant showed smaller fiber volley amplitude (repeated measure two-way ANOVA: genotype effect, *F*_*1,28*_ = 7.995, *P* = 0.0086, *n* = 15 each; Sidak’s test: *P < 0.05, ***P < 0.001). **c** Reduced paired-pulse depression in the mutant mice (repeated measure two-way ANOVA: genotype effect, *F*_*1,12*_ = 22.15, *P* = 0.0005, *n* = 7 each; Sidak’s test: ****P < 0.0001). Sample traces show EPSPs evoked by paired stimulation at the 50 ms interval. Scale bar: 20 ms, 0.2 mV. **d** Significant reduction in the magnitude of synaptic depression during 5 Hz stimulation in the mutant mice (*t*_*8*_ = 2.651, *P* = 0.0292, *n* = 5 each), but no significant change at 1 Hz (*n* = 5 each). Sixty pulses were delivered at 1 or 5 Hz, and the depression ratio was measured for the last 15 pulses. Sample traces are averages of 15 consecutive EPSPs during baseline and 5 Hz stimulation. Scale bar: 10 ms, 0.4 mV. **e** Reduced short-term potentiation after high-frequency tetanic stimulation in the mutant mice (repeated measure two-way ANOVA: genotype × time interaction, *F*_7,63_ = 11.69, *P* < 0.0001; Sidak’s test: **P = 0.0051; +/+: *n* = 5, −/− *n* = 6). Tetanic stimulation (100 Hz, 0.5 s repeated three times) was delivered at time 0. EPSP slopes were averaged at 5 min bin for statistical analysis. Sample traces are averages of 15 consecutive EPSPs during baseline, immediately after tetanus and 35 to 40 min after tetanus. Scale bar: 10 ms, 0.5 mV

Transverse hippocampal slices were prepared from adult mice, and field excitatory postsynaptic potentials (EPSPs) were recorded at the MPP-GC synapse (see Additional file 1 for the detailed method). We first examined the input-output relationship of MPP-GC synaptic transmission by changing the intensity of stimulation. The presynaptic fiber volley amplitude was reduced in the mutant mice, and the EPSP slope showed a trend toward reduction (Fig. 1a and b). The relationship between fiber volley amplitude and EPSP slope was similar between wild-type and mutant mice (Fig. 1b). These results suggest that the presynaptic fiber, MPP, is less excitable in the mutant mice, while the basal synaptic efficacy at the MPP-GC synapse is unaffected. Next we examined several forms of activity-dependent synaptic plasticity. Paired-pulse stimulation induces short-term synaptic depression at this synapse [10]. The mutant mice showed smaller paired-pulse depression at stimulus intervals shorter than 1 s (Fig. 1c). Similarly, synaptic depression during repetitive stimulation was significantly reduced in the mutant mice at 5 Hz, but not at 1 Hz (Fig. 1d). Then we examined the effect of short high-frequency stimulation (HFS). In both wild-type and mutant mice, HFS induced long-term potentiation (LTP) of synaptic transmission. However, initial short-term potentiation (STP) decaying after HFS was significantly smaller in the mutant mice (Fig. 1e). The excitability of MPP during HFS was not significantly changed in the mutant mice (see Supplementary Discussion). Taken together, our results indicate that activity-dependent bidirectional short-term plasticity is attenuated at the MPP-GC synapse in Shn2 knockout mice.

The present study demonstrated that the MPP-GC synaptic efficacy is intact at the basal condition, but less sensitive to neuronal activity in Shn2 knockout mice. We have previously shown that synaptic facilitation during 1 Hz stimulation is strongly reduced at the GC output mossy fiber to CA3 synapse in these mice [4]. Therefore, activity-dependent short-term regulation is impaired at both input and output synapses in the GCs of Shn2 knockout mice, which may underlie impaired hippocampus-dependent cognitive functions in these mice [4].

Paired-pulse depression or facilitation is generally thought to be mediated by presynaptic mechanisms. However, it has been reported that desensitization of α-Amino-3-hydroxy-5-methyl-4-isoxazolepropionic acid (AMPA) receptors also contributes to paired-pulse depression at the MPP-GC synapse [11], which raises a possibility that the reduced synaptic depression observed here represents altered functions of the postsynaptic GCs. LTP at the MPP synapse is induced in an N-methyl-D-aspartate (NMDA) receptor-dependent manner [10] and shares expression mechanisms with the well-characterized Schaffer collateral/commissural fiber-CA1 LTP [12]. Since the STP phase also largely depends on the NMDA receptor-dependent postsynaptic processes [10], the reduced STP may be due to weaker activation of NMDA receptors in Shn2 knockout mice. However, partial block of NMDA receptors is supposed to reduce LTP more severely than STP [13], which is opposite to our present observation. Although a possibility of presynaptic changes cannot be excluded (see Supplementary Discussion), the result of our previous morphological study implies possible postsynaptic mechanisms underlying the reduced STP. We have shown that the dendritic spine neck of the GCs is longer and thinner in Shn2 knockout mice [7]. In CA1, the STP phase has been shown to require lateral diffusion of AMPA receptors from a pre-existing extra-synaptic surface pool [14]. The abnormal spine morphology of GCs may hamper rapid lateral diffusion of AMPA receptors shortly after HFS, thereby preferentially reducing STP. In addition, an immunofluorescent analysis revealed an approximately 50% decrease in expression of the AMPA receptor subunit GluA1 in the dentate molecular layer of Shn2 knockout mice [7]. Given the intact basal transmission at the MPP-GC synapse in these mice, the reduced GluA1 expression may mostly represent a reduced surface pool of extra-synaptic AMPA receptors, which could also contribute to impaired STP. A similar reduction of STP has been demonstrated in the CA1 region of mice with *Disc1* mutation that modeled a risk allele for schizophrenia [15], suggesting that reduced STP could be common synapse pathology in schizophrenia.

The present study has demonstrated the functional defects at the MPP-GC synapse of Shn2 knockout mice that appeared to be in good agreement in part with altered spine morphology previously demonstrated in these mice. These morpho-functional analyses of model mice would provide crucial information in interpreting altered spine morphology in patients with schizophrenia, intellectual disability and other related neuropsychiatric disorders and eventually in understanding essential synapse pathology in these disorders.

## Additional file

Additional file 1: Materials and Methods. Supplementary Discussion. (DOCX 24 kb)

## Abbreviations

AMPA: α-Amino-3-hydroxy-5-methyl-4-isoxazolepropionic acid
EPSP: Excitatory postsynaptic potential
GC: Granule cell
HFS: High-frequency stimulation
HIVEP2: Human immunodeficiency virus type I enhancer binding protein 2
LTP: Long-term potentiation
MPP: Medial perforant path
NMDA: N-methyl-D-aspartate
Shn2: Schnurri-2
STP: Short-term potentiation
