# Supplementary material for: Attenuated bidirectional short-term synaptic plasticity in the dentate gyrus of Schnurri-2 knockout mice, a model of schizophrenia
Source: Mol Brain. 2018 Oct 1;11:56. doi: 10.1186/s13041-018-0400-9 (PMC6167857; doi:10.1186/s13041-018-0400-9)
Supplement: Supplementary file 1 — Materials and Methods. Supplementary Discussion. (DOCX 24 kb) [file 13041_2018_400_MOESM1_ESM.docx]

**Additional file 1**

**Attenuated bidirectional short-term synaptic plasticity in the dentate gyrus of Schnurri-2 knockout mice, a model of schizophrenia**

Katsunori Kobayashi^*^, Tsuyoshi Takagi, Shunsuke Ishii, Hidenori Suzuki,

and Tsuyoshi Miyakawa

*Correspondence to:

Katsunori Kobayashi

Email: kkatsu-tky@umin.ac.jp

**Materials and Methods**

**Animals**

Male Schnurri-2 knockout mice and their wild-type littermates at the age of 15 to 19 weeks were used for experiments. Mice were housed in group in the institutional standard condition (14:10 light/dark cycle; lights on at 6:00 A.M. through 8:00 P.M.) with ad libitum access to food and water. Animal use and procedures were in accordance with the National Institute of Health guidelines and approved by the Animal Care and Use Committee of Nippon Medical School.

**Electrophysiological analysis**

Mice were decapitated under deep halothane anesthesia, and both hippocampi were isolated. Transverse hippocampal slices (300 μm) were cut using a tissue slicer in an ice-cold saline composed of (in mM): NaCl, 125; KCl, 2.5; NaH_2_PO_4_, 1.0; NaHCO_3_, 26.2; glucose, 11; CaCl_2_, 2.5; MgCl_2_, 1.3 (equilibrated with 95% O_2_ / 5% CO_2_). Slices were then incubated for 30 min at 30 °C and maintained in a humidified interface holding chamber at 25 - 27 °C before recordings. Electrophysiological recordings were made in a submersion-type chamber maintained at 27.0 - 27.5 ºC and superfused at 2 ml/min with the saline. For recording excitatory postsynaptic potentials (EPSPs) arising from the medial perforant path-granule cell synapse, a glass recording pipette filled with 2 M NaCl and bipolar stimulating electrodes were placed in the middle third of the molecular layer in the dentate gyrus. The initial slope of EPSPs was measured on analysis. Single electrical stimulation was delivered at a frequency of 0.05 Hz unless otherwise specified. The input-output relationship was examined in the normal saline, and other experiments were performed in the presence of 100 μM picrotoxin (Wako Pure Chemical Industries, Ltd., Osaka, Japan). Long-term potentiation was induced by high-frequency stimulation (HFS: 100 Hz, 0.5 s) repeated three times at an interval of 20 s. To examine excitability of presynaptic fibers during HFS, the averaged fiber volley amplitude of 5 responses around the end of each train was compared with that of the baseline responses. All recordings were made using a Multiclamp 700B amplifier (Molecular Devices, Sunnyvale, CA, USA), filtered at 2 kHz and stored in a personal computer via an interface (digitized at 10 kHz).

**Statistics**

All data are presented as means ± s.e.m. Statistical tests were performed using GraphPad Prism version 7.01. The data were analyzed by the unpaired two-tailed Student’s t test or repeated measure two-way ANOVA followed by the Sidak’s test. Statistical significance was set at *P* < 0.05. The number of data “*n*” represents the number of slices.

**Supplementary Discussion**

There remains a possibility that reduced short-term potentiation (STP) observed in the mutant mice was caused by presynaptic changes. While the fiber volley amplitude tended to decrease during HFS (wild type: 90.1 ± 10.1% of baseline, n = 5; mutant: 92.3 ± 8.5 % of baseline, n = 6), there was no significant difference between the genotypes. Therefore, a change in the excitability of presynaptic fibers during HFS is unlikely to explain the reduced STP. HFS can induce rapidly decaying post-tetanic potentiation (PTP) that is formed by presynaptic induction and expression mechanisms. At the medial perforant path-granule cell synapse, HFS delivered in the presence of an N-methyl-D-aspartate (NMDA) receptor antagonist induces small PTP-like synaptic potentiation that decays within 5 min [10]. Therefore, we cannot exclude a possibility that altered presynaptic potentiation contributed to the reduction of STP especially during the initial few minutes. An increase in the basal transmitter release probability can reduce presynaptic forms of potentiation. However, since the increased basal transmitter probability also increases presynaptic short-term depression such as paired-pulse depression, it cannot solely explain our present results. Presynaptic mechanisms underlying the formation of PTP might be impaired in the mutant mice. To solve this issue, a detailed analysis of HFS-induced synaptic potentiation in the presence of the NMDA antagonist would be required.
